# Radiation enteropathy and leucocyte-endothelial cell reactions in a refined small bowel model

**DOI:** 10.1186/1471-2482-4-10

**Published:** 2004-09-13

**Authors:** Louis Banka Johnson, Amjid Ali Riaz, Diya Adawi, Lena Wittgren, Sven Bäck, Charlotte Thornberg, Nadia Osman, Virgil Gadaleanu, Henrik Thorlacius, Bengt Jeppsson

**Affiliations:** 1 Department of Surgery, Malmö University Hospital, Lund University, Malmö, Sweden; 2 Imperial College School of Medicine, Hammersmith Hospital, London, United Kingdom; 3 Department of Radiation Physics, Malmö University Hospital, Lund University, Malmö, Sweden; 4 Dept. of Food Technology, Lund University, Lund, Sweden; 5 Department of Pathology, Malmö University Hospital, Lund University, Malmö, Sweden

## Abstract

**Background:**

Leucocyte recruitment and inflammation are key features of high dose radiation-induced tissue injury. The inflammatory response in the gut may be more pronounced following radiotherapy due to its high bacterial load in comparison to the response in other organs. We designed a model to enable us to study the effects of radiation on leucocyte-endothelium interactions and on intestinal microflora in the murine ileum. This model enables us to study specifically the local effects of radiation therapy.

**Method:**

A midline laparotomy was performed in male C57/Bl6 mice and a five-centimetre segment of ileum is irradiated using the chamber. Leucocyte responses (rolling and adhesion) were then analysed in ileal venules 2 – 48 hours after high dose irradiation, made possible by an inverted approach using intravital fluorescence microscopy. Furthermore, intestinal microflora, myeloperoxidase (MPO) and cell histology were analysed.

**Results:**

The highest and most reproducible increase in leucocyte rolling was exhibited 2 hours after high dose irradiation whereas leucocyte adhesion was greatest after 16 hours. Radiation reduced the intestinal microflora count compared to sham animals with a significant decrease in the aerobic count after 2 hours of radiation. Further, the total aerobic counts, *Enterobacteriaceae and Lactobacillus *decreased significantly after 16 hours. In the radiation groups, the bacterial count showed a progressive increase from 2 to 24 hours after radiation.

**Conclusion:**

This study presents a refinement of a previous method of examining mechanisms of radiation enteropathy, and a new approach at investigating radiation induced leucocyte responses in the ileal microcirculation. Radiation induced maximum leucocyte rolling at 2 hours and adhesion peaked at 16 hours. It also reduces the microflora count, which then starts to increase steadily afterwards. This model may be instrumental in developing strategies against pathological recruitment of leucocytes and changes in intestinal microflora in the small bowel after radiotherapy.

## Background

Radiotherapy is widely used in treating different types of cancer and is an effective therapeutic modality against abdominal and pelvic cancers. Gastrointestinal tract damage by radiotherapy limits its efficacy in cancer treatment. The small bowel is highly radiosensitive and very mobile and is thus an important dose-limiting organ during radiation therapy for abdominal and pelvic cancer [1]. Radiation induces an inflammatory response in target and surrounding tissues, which is characterised by accumulation of plasma proteins and leucocytes. Leucocyte recruitment is a multi-step process, which includes leucocyte rolling, activation and firm adhesion to the endothelium [2]. Leucocyte rolling reduces the velocity and allows time for leucocytes to detect chemotactic signals on the endothelial surface. It is now widely accepted that leucocyte rolling is a precondition for the subsequent firm adhesion and extravascular accumulation [3,4]. With intravital microscopy, several studies have demonstrated that the selectin family of adhesion molecules predominantly mediates leucocyte rolling and that stationary adhesion is supported by the β_2_-integrins [5,6].

Several animal models exist in order to investigate radiation-induced leucocyte endothelium cell responses, which can broadly be divided into two groups. Topical radiation (abdominal and pelvic) [7-9] and more recently, segmental radiation of an isolated short segment of small intestine where different responses to radiation are examined [1,10]. We refined and developed the latter model, incorporating a platform where mice can be placed allowing exposure of the required segment of intestine for irradiation. This allows us to expose exteriorised intestinal sections to tailored high dose radiation, greatly minimizing scattering effects and thereby consequently avoiding surrounding tissue damage.

The purpose of this study was to refine a small bowel radiation model in order to study the time responses in terms of leucocyte rolling, adhesion, myeloperoxidase (MPO) levels, histology and intestinal floral changes in response to high dose radiation of the ileum, where the exact biologically effective dose could be calculated.

## Methods

### Animals

Male C57Bl/6J mice weighing 22–26 g were kept under standard laboratory conditions maintained on a 12 hour light and 12 hour dark cycle and were allowed free access to animal chow and tap water *ad libitum*. All experimental procedures were performed in accordance with legislation on the protection of animals and were reviewed and approved by the Lund University Ethic's Committee for Animal Experimentation.

### Anesthetic and surgical preparation

**Figure 1:**
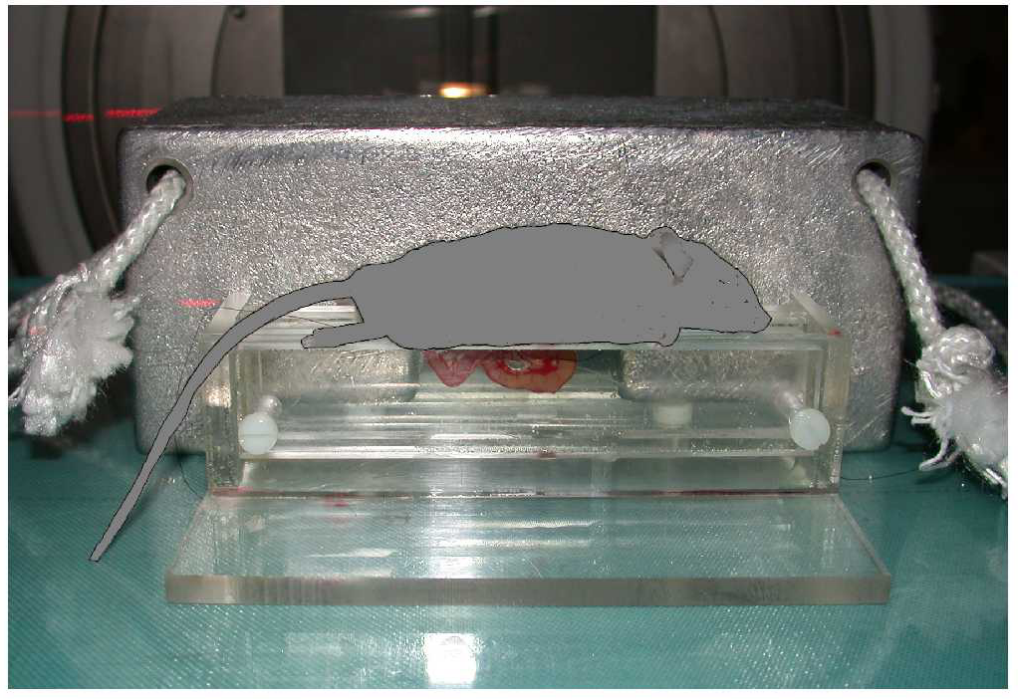
The chamber for segmental intestinal radiation exposure.

The mice were anesthesized with 7.5 mg Ketamine hydrochloride (Hoffman-La Roche, Basel Switzerland) and 2.5 mg Xylazine (Janssen Pharmaceutica, Beerse, Belgium) per 100 g body weight by intraperitoneal (*i.p*.) injection. The animals were placed in supine position on a heating pad (37°C) for maintenance of body temperature. A small midline incision (1.0–1.5 cm) was performed and a 5 cm segment of ileum located 5 cm from the ileocaecal valve was exteriorised and marked with 5-0 non-absorbable sutures. Any other visible prolapsed abdominal content was replaced back into the abdomen and the animal was placed on the specially designed frame/chamber (Figure 1), with the loop of intestine fixed between two perspex sheets. The exposed ileum was subjected to a single dose of high dose radiation of 19 Gy and thereafter replaced in the abdomen and the incision closed with a polypropylene suture. At the appropriate time a polyethylene catheter (PE-10 with an internal diameter of 0.28 mm) was placed into the internal jugular vein for administration of fluorescent markers. Leucocyte-endothelium interactions were then observed using an inverted intravital fluorescence microscopy (IIVM) at different time points (2–48 hours).

### Experimental protocol

The animals were divided into two different groups; Radiation & Surgery group (R+) and Sham Radiation & Surgery group (R-) which served as negative controls. The irradiated groups of mice (*n *= 6/time point) were exposed to 19 Gy of radiation and leucocyte-endothelium interactions were measured 2, 6, 16, 24 and 48 hours after induction of radiation using an IIVM (each group *n *= 6, likewise the R- groups). At the end of the procedure samples were collected for histology, and measurement of intestinal microflora, MPO and systemic leukocyte counts.

### Radiation

The irradiations were undertaken using a clinical linear accelerator (Varian Clinac 2100C). The exteriorised mouse intestine was positioned between Perspex slabs to accomplish sufficient secondary radiation scatter and thereby a reproducible and homogenous dose distribution. The absorbed dose was verified with independent measurements and was found to be within 5% throughout the intended volume using this technique. Using an asymmetrically half blocked 6 MV beam and extra lead shielding (Figure 1), the treatment field perfectly fitted the exteriorised intestine while the remaining body was kept outside the radiation beam. An absorbed dose of 19 Gy was delivered to the intestine as this dose causes consistent structural, cellular, and molecular changes [11]. The absorbed dose rate was 3.2 Gy/minute and consequently the irradiation time for each animal was approximately 6 minutes. During irradiation the intestine in the chamber is protected from large temperature variations and trauma by perspex sheets. The exposure time from surgery, through irradiation to wound closure is kept at a minimum, taking approximately 15 minutes, thus keeping stress and trauma levels low.

### Intravital microscopy

Observations of the intestinal microcirculation were made using an inverted Olympus microscope (IX70, Olympus Optical Co. GmbH, Hamburg Germany) equipped with different lenses (x10/NA 0.25 and x40/NA 0.60). The microscopic images were televised using a charge-coupled device video-camera (FK 6990 Cohu, Pieper GmbH, Schwerte, Germany) and recorded on videotape (Sony SVT-S3000P S-VHS recorder) for subsequent off-line analysis. To prevent drying during microscopic observations the intestinal segment was placed on a saline moistened cotton gauze and thereafter positioned under the microscope. After a 5-min equilibration period, quantitative measurements were taken. Analysis of leucocyte-endothelium interactions (rolling and adhesion) was made in venules (inner diameter 15–30 μm) with stable resting blood flow. Blood perfusion within individual microvessels was studied after contrast enhancement by *i.v*. administration of fluorescein isothiocyanate (FITC)-labelled dextran (MW 150000), (0.05 ml, 5 mg/ml, Sigma Chemical Co. St. Louis, MO, U.S.A.). *In vivo *labelling of leucocytes with rhodamine-6G (0.1 ml, 0.5 mg/ml, Sigma Chemical Co. St. Louis, MO, U.S.A.) enabled quantitative analysis of leucocyte flow behavior in the ileum microcirculation. Due to its relatively higher molecular weight FITC-dextran stains/labels the intravascular plasmatic phase of the blood under epi-illumination with blue light (excitation wavelength 490 nm; emission wavelength 510 nm) whereas the lower molecular weight of Rhodamine 6G allows for labelling of leucocytes and platelets using green fluorescent light (excitation wavelength 530 nm; emission wavelength 560 nm). Quantification of microcirculatory parameters was performed off-line by frame-to-frame analysis of the videotaped images. Leucocyte rolling was determined by counting the number of leucocytes passing a reference point in the venule per 20 sec and is expressed as cells/min. Firm adhesion was measured by counting the number of cells adhering to the venular endothelium (200–300 μm long segments) and remained stationary for 20 sec and is given as cells/mm venule length. Blood flow velocities were analysed by means of a video assisted computer image analysis programme, CapImage software (Zeintl, Heidelberg, Germany). The staining of the plasmatic phase by FITC-dextran gives an indirect enhancement of red blood cells which appear dark in the illuminated surrounding plasma. The CapImage uses the FITC-dextran image to calculate the red blood cell velocity. The velocity was calculated as a mean value from 5–8 measurements per venule and is expressed as mm/sec. Venular wall shear rate was determined based on the Newtonian definition: wall shear rate = 8 [(red blood cell velocity/1.6)/venular diameter] as described previously [12].

### MPO measurement

The enzyme myeloperoxidase (MPO) is abundant in neutrophil leucocytes and has been found to be a reliable marker for the detection of neutrophil accumulation in inflamed tissue. To determine tissue MPO content, radiated ileal tissue was collected, weighed, homogenized in 10 ml 0.5% hexadecyltrimethylammonium bromide, and freeze thawed, after which the MPO activity of the supernatant was assessed. The enzyme activity was determined spectrophotometrically as the MPO-catalysed change in absorbance occurring in the redox reaction of H_2_O_2 _(460 nm, 25°C). Values are expressed as MPO units per g tissue.

### Histological study

Samples from the irradiated small intestine were placed in 4% phosphate buffered formaldehyde. Paraffin-embedded samples were sliced and studied under light microscopy after staining with hematoxylin and eosin. At least 3 slides were studied from each specimen in a blinded fashion.

### Intestinal microflora

Tissue samples from the irradiated small intestine were first placed in 5 ml of sterile transport medium [13]. Samples were then placed in an ultrasonic bath (Millipore, Sweden) for 5 minutes and then rotated on Chiltern (Terma-Glas, Gothenberg, Sweden) for 2 minutes. After a conventional dilution procedure, viable counts were obtained from Brain Heart Infusion (BHI) that was incubated aerobically and anaerobically at 37°C for 72 hours (aerobic and anaerobic bacterial count, respectively), and from Rogosa agar (Oxoid, Hampshire, England) that was incubated anaerobically at 37°C for 72 hours (lactobacilli counts). Viable counts were also obtained from violet red-bile-glucose agar (VRBD) (Oxoid, Hampshire, England) that was incubated aerobically at 37°C for 24 hours (Enterobacteriaceae counts) and from BHI agar containing gram-negative anaerobic supplement (Oxoid, Hampshire, England) that was incubated anaerobically at 37°C for 72 hours (gram negative anaerobic bacterial counts).

### Systemic leucocyte counts

20 μl blood was mixed with Turk's solution (0.2 mg gentian violet in 1 ml glacial acetic acid, 6.25 % v/v) in a 1:10 dilution. Leucocytes were counted and differentiated as polymorphonuclear (PMNL) or mononuclear (MNL) cells in a Burker chamber.

### Statistical analysis

Statistical evaluations were performed using the Kruskal-Wallis one way analysis of variance on ranks for unpaired samples (Dunn's post hoc test was used). For bacterial microflora in comparing 2 groups we used Mann-Whitney Rank sum test, and for the comparison of the different time points within the radiated groups we used One Way ANOVA followed by multiple comparisons versus control group (Dunnett's method). The results are presented as mean values ± SEM. Differences were considered to be significant at *P *< 0.05.

## Results

### Radiation-induced leucocyte-endothelium interactions in the ileum

**Figure 2:**
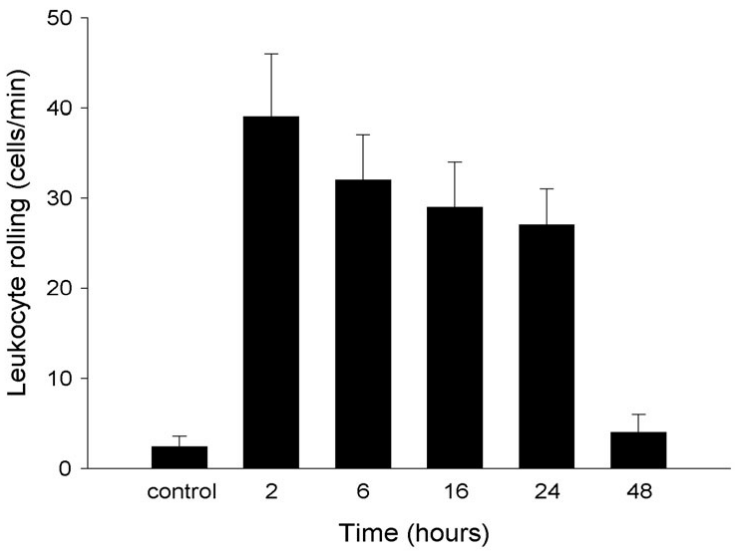
Venular leucocyte rolling in the mouse ileum at different time points after radiation. Data represents mean ± SEM.

**Figure 3:**
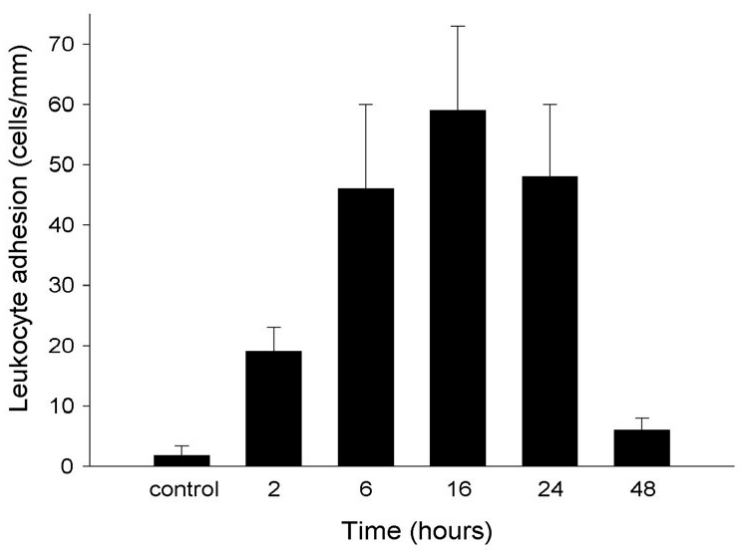
Venular leucocyte adhesion in the mouse ileum at different time points after radiation. Data represents mean ± SEM.

Intravital microscopic studies in post-capillary venules of the distal ileum in sham operated mice (controls) revealed only occasional interactions between leucocytes and the microvascular endothelium, *i.e*. the number of rolling and adherent leucocytes was 2.4 ± 1.2 cells/min and 1.7 ± 1.7 cells/mm, respectively. In contrast, radiation (19 Gy) evoked a marked time-dependent leucocyte response, *i.e*. a significant increase in both leucocyte rolling and firm adhesion over time (Figures 2 and 3, *P *< 0.05, *vs*. controls, *n *= 5–10). We observed that leucocyte rolling peaked two hours after radiation (38 ± 7 cells/min, (Figure 2), *P *< 0.05 *vs*. sham, *n *= 6–10), whereas leucocyte adhesion was maximum after 16 hours showing a marked response of 59 ± 14 cells/mm (Figure 3, *P *< 0.05 *vs*. sham, *n *= 6). Interestingly, both the leucocyte rolling and adhesion responses to radiation returned to baseline levels 48 hours after radiation (Figures 2 and 3, *P *> 0.05, *vs*. sham, *n *= 5–10). There was no difference in the hemodynamic parameters between the different experimental groups (Table 1) and also no significance difference could be seen in the systemic leucocyte counts.

**Table 1:** Hemodynamic parameters in ileal venules

	***Diameter ***(μm)	Red blood cell velocity (mm s^-1^)	Wall shear rate (s^-1^)
Sham 2 hrs	25.5 ± 2.1	1.57 ± 0.21	314 ± 44
Sham 16 hrs	26.2 ± 1.2	1.61 ± 0.14	309 ± 19
Radiation 2 hrs	22.2 ± 1.6	0.93 ± 0.08	212 ± 44
Radiation 6 hrs	24.5 ± 2.4	1.53 ± 0.12	319 ± 56
Radiation 16 hrs	21.7 ± 2.5	0.93 ± 0.22	222 ± 74
Radiation 24 hrs	20 ± 1.9	1.26 ± 0.16	408 ± 48
Radiation 48 hrs	25.5 ± 2.3	1.32 ± 0.11	264 ± 57

Radiation was directed to the ileum and leukocytes responses were measured after 2–48 hours. Sham-operated controls underwent identical procedures except undergoing radiation. Responses measured at 2 and 16 hours (n = 5,6). Blood flow velocities were measured off-line by frame-to-frame analysis of the videotaped images. Data are mean ± SEM.

### Histological changes following radiotherapy

**Figure 4:**
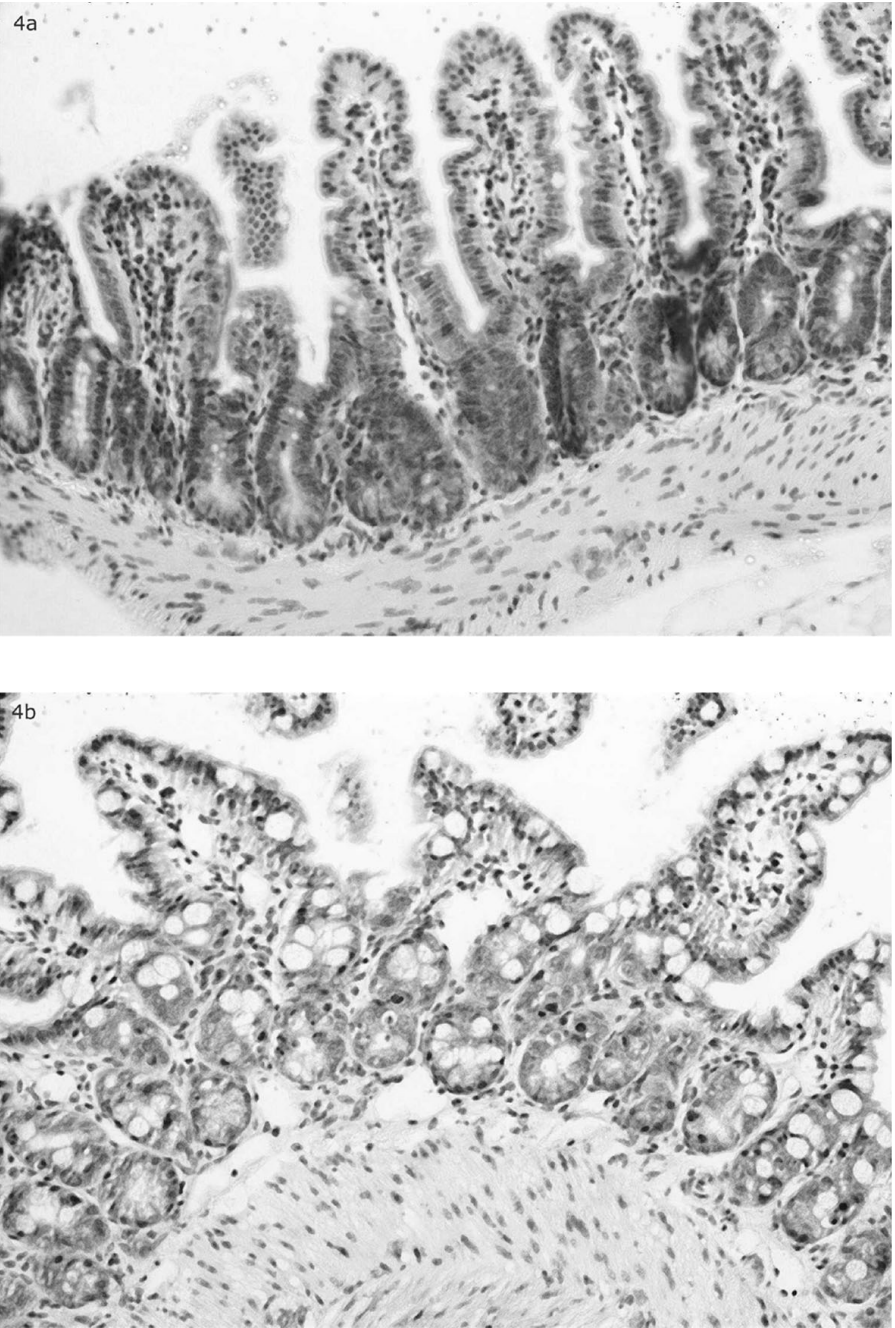
**A **Cross section of intestinal wall 2 hrs after irradiation. No marked differences in the number of inflammatory cell types compared to the controls. **B **Cross section of intestinal wall 48 hrs after irradiation. A vast increase of goblet and apoptotic cells was seen in the whole length of the epithelium and crypts. There was a reduction in lymph vessel ectasia, oedema and in the number of inflammatory cells present compared to earlier time points.

**Figure 5:**
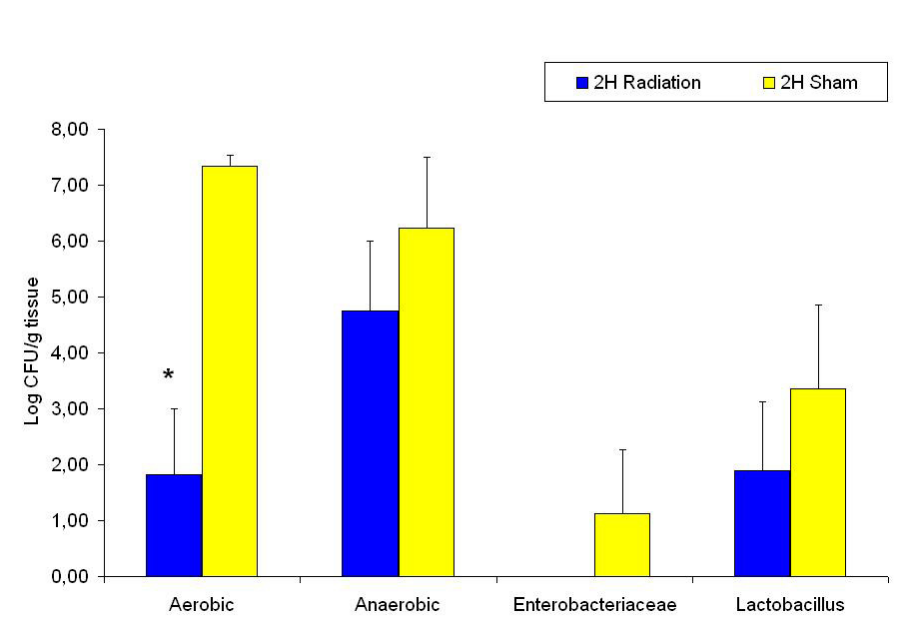
Ileum bacterial microflora in sham and 2 hours after radiation groups. * denotes p < 0.05 compared to sham group.

At 2 hours we could not observe any marked differences in the number of inflammatory cell types compared to the controls (Figure 4). At 6 hours we found quite a number of apoptotic epithelial cells, and a few inflammatory cells – mainly neutrophil granulocytes in the lamina propria. Both the granulocytes and the apoptotic cells increased in numbers at 16 hours. An increase in the inflammatory infiltrate was also observed in the smooth muscle layer (muscularis propria). 24 hours after radiation the muscularis mucosae was oedematous and infiltrated by granulocytes; there was clearly visible lymph vessel ectasia and apoptosis mainly in the deeper parts of the crypts. Forty-eight hours after radiation a vast increase of goblet and apoptotic cells was seen in the whole length of the epithelium and crypts. On the other hand there was a reduction in lymph vessel ectasia, oedema and in the number of inflammatory cells present (Figure 5).

### Intestinal microflora

**Figure 6:**
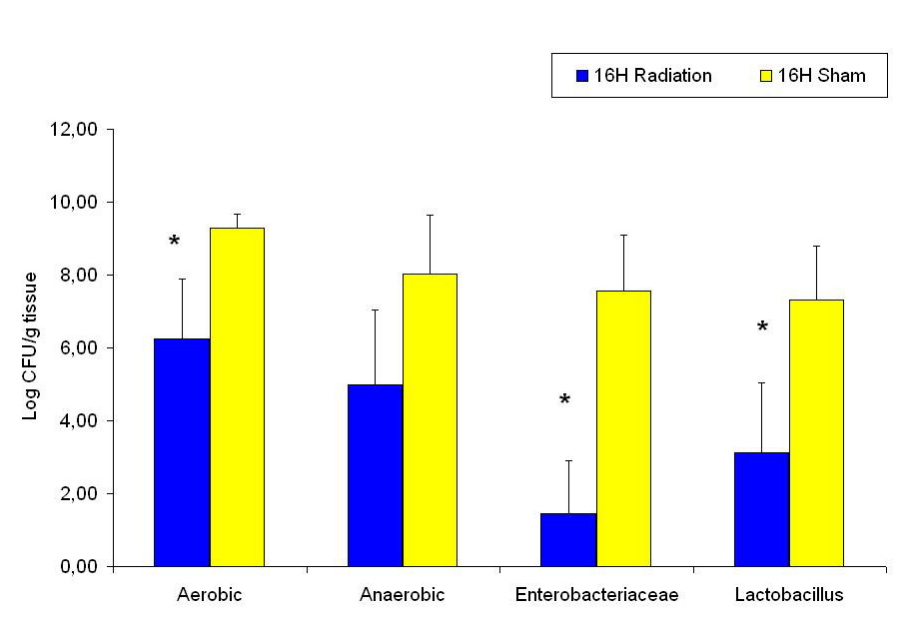
Ileum bacterial microflora in sham and 16 hours after radiation groups. * denotes p < 0.05 compared to sham group.

**Figure 7:**
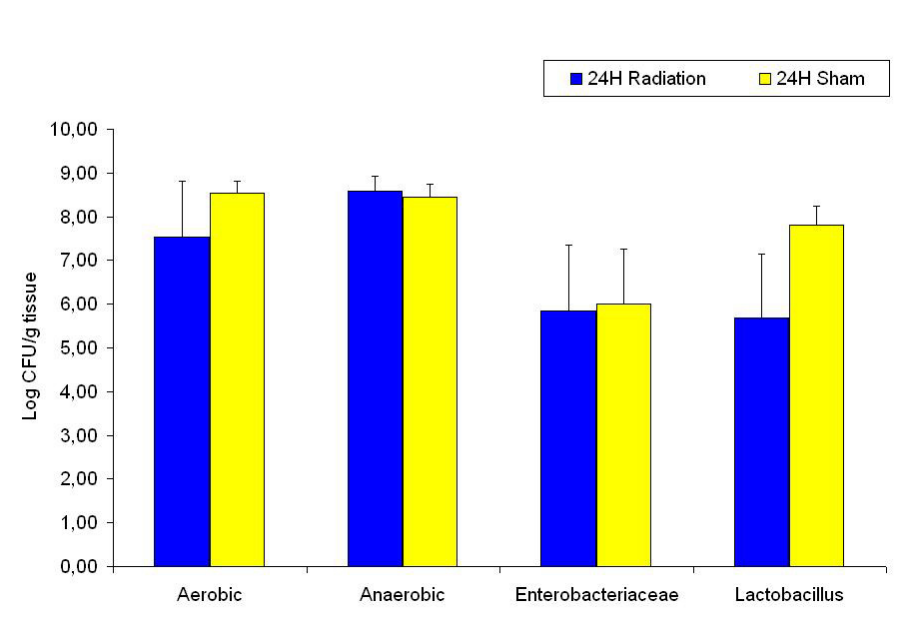
Ileum bacterial microflora in sham and 24 hours after radiation groups. No significant difference between the experimental groups.

**Figure 8:**
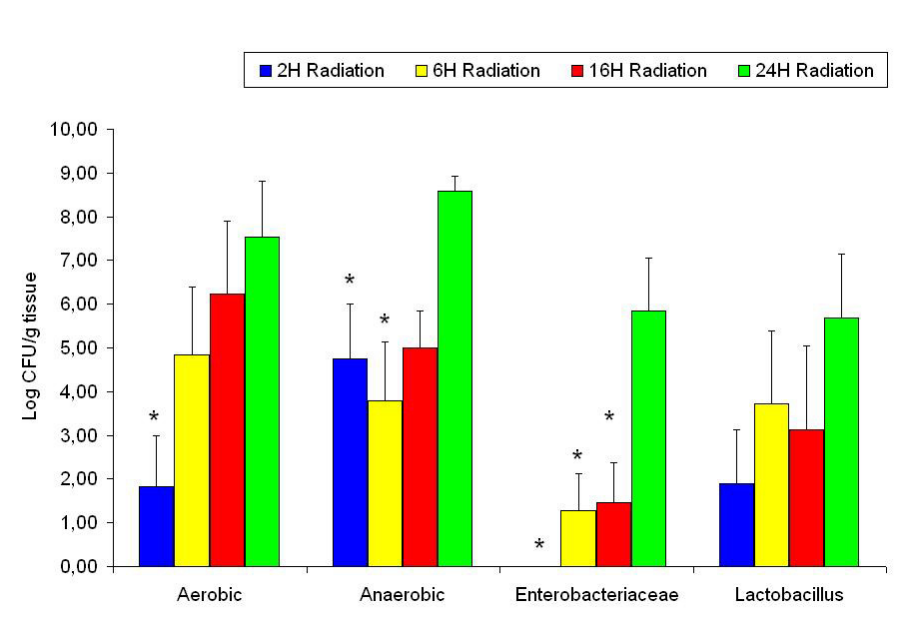
Ileum bacterial microflora in the radiated groups at different time points. * denotes p < 0.05 compared to 24 hours radiated group.

Compared to the sham groups; the aerobic, *Enterobacteriaceae*, *Lactobacillus *and anaerobic counts had decreased two hours after radiation (Figure 5), the same groups, with the exception of the anaerobic count were significantly decreased sixteen hours after radiation (Figure 6). There were no significant differences between the experimental groups twenty four hours after radiation compared to the sham group (Figure 7). When assessing the trends within the various radiated bacterial groups compared to the 24 hour levels we found significant decreases in the aerobic count at 2 hours; in the anaerobic count at 2 and 6 hours; and in the *Enterobacteriaceae *at 2, 6 and 16 hours. There were no significant changes in the *Lactobacillus *count at the different time points within the radiated groups (Figure 8).

### MPO measurement

There were no differences in MPO measurements in the experimental groups.

## Discussion

The frequent use of radiotherapy for abdominal and pelvic malignancies results in an increased risk of radiation enteritis [14]. The dose of radiation that can be applied in clinical practice is usually limited by the need to restrict the number and severity of side effects in normal tissues surrounding a tumour, which are unavoidably exposed to radiation [8]. Intestinal radiation toxicity (radiation enteropathy) is characterised by mucosal barrier breakdown and inflammation, followed by development of progressive vascular sclerosis and intestinal wall fibrosis. The process is accompanied by sustained over expression of inflammatory and fibrogenic cytokines [15,16].

An early inflammatory response, beginning a few hours after irradiation, characterised by leucocyte infiltration into the irradiated organs is regarded as one of the main determinants of radiation-induced organ damage [17,18]. The development of an inflammatory response involves sequential leucocyte-endothelial cell interactions. Different families of cell adhesion molecules have been shown to participate in the process of leucocyte recruitment [19]. There are three major families of adhesion molecules involved in the leucocyte recruitment process, the selectins, the integrins and the immunoglobulin supergene families [20].

The present study has concentrated on the acute effects of radiation injury on leucocyte rolling and adhesion at specific time points after radiation. We found that radiation evoked a marked time dependent leucocyte response with a significant increase in both leucocyte rolling and firm adhesion over time. Leucocyte rolling peaked 2 hours after radiation whereas leucocyte adhesion was highest after 16 hours showing a marked response. Interestingly, both the leucocyte rolling and adhesion responses to radiation were back to baseline 48 hours after radiation. An intravital microscopic study of radiation-induced leucocyte-endothelial cell interaction using abdominal radiation and a dose of 20 Gy revealed an increased leucocyte rolling in mesenteric venules 2 hours after radiation, with a marked increase in leucocyte adhesion and emigration noted at 6 hours [18]. In another study of radiation-induced inflammatory damage, abdominal irradiation was administered using 4 and 10 Gy respectively [8]. Here an increase in leucocyte rolling was observed 2 hours after radiation, which then returned to basal levels at 6 and 24 hours respectively. An increase in leucocyte adhesion was also observed 2 hours after irradiation, which was then sustained during the 24 hour observation period [8]. In our study we showed the maximum effect on rolling after 2 hours and adhesion after 16 hours and the return to basal levels 48 hours after radiation. We used a single high dose radiation of 19 Gy directly to an exteriorised segment of ileum. This dose was chosen because it has been shown to give a good correlation or dose response relationship of histopathological changes (e.g. mucosal ulceration, vascular sclerosis) to incidence of clinical complications and cellular evidence of injury [1]. When comparing our results (ileal venule measurements) to those from other tissue, namely from the pial venules of cerebral microvasculature of the rat after 20 Gy irradiation [21], we find that the results follow a similar time course. Assuming that the radiation dose distribution is similar in all experiments mentioned above, the differences in peak times for leucocyte rolling and adhesion may probably be due to differences in radiation dose/duration, the extent of trauma, the effect of anaesthesia, the mode and duration of experiments.

Endogenous bacterial flora produces nutrients (e.g. short-chain fatty acids) for the mucosa; prevents overgrowth of potentially pathogenic micro-organisms; stimulates the immune system especially the gut-associated lymphoid tissue; helps eliminate toxins from the lumen and participates in intestinal regulation, motility and blood flow [22]. Radiation on the other hand influences and alters the mucosal microflora, and this in combination with barrier dysfunction leads to a translocation of microbes through the mucosa into blood circulation [23]. Our experiment shows that radiation affects the intestinal microflora. Two hours after radiation the aerobic, anaerobic, *Enterobacteriaceae *and *Lactobacillus *counts were decreased and after 16 hours the aerobic, *Enterobacteriaceae *and *Lactobacillus *counts were still decreased in the radiated groups compared to sham controls. Twenty-four hours after radiation there was no significant difference between the experimental groups. Comparing the results of the irradiated groups alone, one observes an increase in bacterial count over time after radiation. It seems that radiation decreases the bacterial count at early time points with no difference in total bacterial count at late time points. This total count does not reflect the difference in bacterial species within each group, and thus, further investigations are needed to study the imbalances that occur. One study has shown that microorganisms such as Escherichia, Proteus, Clostridium, normally absent in healthy animals, appear in the intestines of guinea pigs subjected to irradiation. At the same time lactobacilli and bifidobacteria sharply decrease in number [24]. Bacterial overgrowth and intestinal pseudo-obstruction may succeed abdominal radiotherapy and the impaired motility emerges as a causal factor for gastrointestinal colonization with gram-negative bacilli. Abnormal motility and gram-negative bacilli in the gut may be essential in the pathogenesis of late radiation enteropathy [25]. Changes in intestinal microflora therefore most probably affect the course of the development of radiation enteropathy. Acute intestinal symptoms during pelvic radiotherapy may not depend only on mucosal damage [26]. Post-radiation gut structural damage occurs early and parallels functional changes of the intestinal mucosa, including increased epithelial permeability (shown both in vivo and ex vivo), activation of secretory pathways, decreased nutrient absorption, diarrhoea, and weight loss [27]. The microfloral changes, which we have shown, could play an important role in the structural and functional intestinal changes after radiation, particularly in the presence of intestinal mucosal changes and increased intestinal permeability. Patients with carcinoma of the uterine cervix or endometrium receiving postoperative radiation therapy have a significant decrease in intestinal microflora after the first radiation exposure, whereas at the end of radiotherapy all bacteria have increased and reached basal values except *Enterococcus faecium *1, lactobacilli and total anaerobes. In some patients an overgrowth of some Clostridium spp. (potential pathogens) associated with clinical symptoms, was observed. Patients receiving radiotherapy may thus benefit from the intake of oral bacteriotherapy [28]. The importance of investigating the effects of radiation on the different bacterial species within the total count is therefore of significance for the modulation of treatment regimes.

The histological changes following radiation are both time and dose dependent [29,30]. Soon after radiotherapy we observed an increase in inflammatory cell-infiltrate, apoptosis, mucin producing goblet cells and oedema, representing the morphological expression of an unspecific reactive process with a supposed protective function. Variations of these changes have been previously observed in the clinical situation. The vast increase in goblet cells that we observed may resemble that seen in necrotising enterocolitis. A resemblance to chronic idiopathic inflammatory bowel disease, eosinophilic colitis and microscopic colitis can also be seen if the mild crypt distortion or withering that occurs with radiation injury is confused with proper crypt architectural distortion of inflammatory disease. Isolated crypts due to nuclear regenerative changes may also mimic the microadenomas of familial adenomatosis polyposis [30]. Histological changes in the pre-existing normal mucosa following preoperative radiotherapy need to be appreciated by the histopathologist if we are to avoid erroneous concurrent diagnosis [30]. Furthermore, a correct assessment of the effects of new treatment regimes or prophylaxis is based on a sound histological judgment.

No differences MPO values could be seen between the controls and the radiated groups. This is probably because it is a crude method of measurement and thus may not be sensitive enough to detect early changes of inflammation.

This study therefore presents a refinement of previous methods of examining effects of radiation enteropathy, and a new approach at investigating radiation induced leucocyte responses in the ileal microcirculation. This new model may be instrumental in developing strategies against pathological recruitment of leucocytes and changes in intestinal microflora in the small bowel.

## Competing interests

None declared.

## Authors' contributions

LBJ designed the study and participated in construction of the chamber. Performed experimental studies and drafted the manuscript.

AAR performed experimental studies and drafted the manuscript.

DA participated in the design of the study and construction of the chamber. Performed experimental studies, drafted the manuscript and performed the statistical analysis.

LW participated in the radiological design of the study, construction of the chamber and the implementation of radiotherapy.

SB participated in the radiological design of the study, chamber and the implementation of radiotherapy.

CT participated in the implementation of radiotherapy.

NO carried out bacteriological studies.

VC performed the histological analysis.

HT assisted with issues related to intravital microscopy.

BJ conceived of the design, participation in construction of the chamber, co-ordination of the study as well as supervision and draft of the manuscript.

## Pre-publication history

The pre-publication history for this paper can be accessed here:


